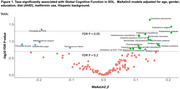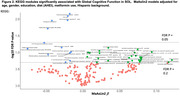# Gut Microbiome Multi‐Omics and Cognitive Function in a Large Latino Cohort

**DOI:** 10.1002/alz.092828

**Published:** 2025-01-09

**Authors:** Natalia Palacios, Scot Gordon, Tao Wang, Curtis Huttenhower, Hector M Gonzalez, Rob Knight, Charles Decarli, Martha L Daviglus, Melissa Lamar, Wassim Tarraf, Jianwen Cai, Robbie Burk, Quibin Qi, Robert C. Kaplan

**Affiliations:** ^1^ Univ. of MA, Lowell, Lowell, MA USA; ^2^ Albert Einstein College of Medicine, Bronx, NY USA; ^3^ Harvard T. H. Chan School of Public Health, Boston, MA USA; ^4^ University of California San Diego, San Diego, CA USA; ^5^ University of California, San Diego, La Jolla, CA USA; ^6^ Department of Bioengineering, University of California San Diego, La Jolla, CA USA; ^7^ Department of Pediatrics, University of California San Diego, La Jolla, CA USA; ^8^ Department of Computer Science & Engineering, University of California San Diego, La Jolla, CA USA; ^9^ Center for Microbiome Innovation, University of California San Diego, La Jolla, CA USA; ^10^ Alzheimer's Disease Research Center, University of California Davis, Sacramento, CA USA; ^11^ University of Illinois at Chicago, Chicago, IL USA; ^12^ Rush Alzheimer's Disease Center, Chicago, IL USA; ^13^ Wayne State University, Detroit, MI USA; ^14^ University of North Carolina, Chapel Hill, NC USA; ^15^ Einstein College of Medicine, Bronx, NY USA; ^16^ Fred Hutchinson Cancer Center Public Health sciences Division, Seattle, WA USA

## Abstract

**Background:**

Few large microbiome studies on Alzheimer’s Disease and Related Dementia (AD/ADRD) have been conducted, especially among US Latinos. We conducted a study within the Study of Latinos‐ Investigation of Neurocognitive Aging (SOL‐INCA) cohort to examine the role of the gut microbiota in cognitive function.

**Methods:**

We analyzed the fecal metagenomes of 2,470 SOL‐INCA participants to, cross‐sectionally, identify microbial taxonomic and functional features associated with cognitive function. Global cognition was defined as an aggregate score based on a cognitive battery (executive function, working memory, among others). Omnibus (PERMANOVA) and feature‐wise analyses (MaAsLin2) were conducted to identify microbiome‐cognition associations, and specific microbial species and pathways (Kyoto Encyclopedia of Genes and Genomes (KEGG modules) associated with cognition. We assessed the accuracy of a Random Forest classifier to distinguish SOL participants with the best (>=1SD above mean) vs. the worst (>=1SD below mean) cognition. We also tested the association of identified taxa and KEGG modules with concurrently collected serum metabolites.

**Result:**

We identified several taxa and pathways significantly associated with cognitive function in SOL. B. longum was the taxa most strongly associated with worse cognition, whereas Eubacterium species (E. siraeum and E.eligens), were associated with better cognition. Several KEGG modules, most strongly Ornithine and Serine biosynthesis, were associated with worse cognition. A microbiome species‐based Random Forest classifier had moderate accuracy (AUC = 0.62) to discriminate between high (1SD or more above mean) vs low (1SD or more below mean) cognition.

**Conclusion:**

In a large Latino cohort, we identified several microbial taxa and KEGG pathways associated with cognition, further implicating the microbiome in AD/ADRD risk.